# Single-Pixel MEMS Imaging Systems

**DOI:** 10.3390/mi11020219

**Published:** 2020-02-20

**Authors:** Guangcan Zhou, Zi Heng Lim, Yi Qi, Guangya Zhou

**Affiliations:** Micro and Nano Systems Initiative, Department of Mechanical Engineering, National University of Singapore, Singapore 117575, Singapore; E0115906@u.nus.edu (G.Z.); limziheng@nus.edu.sg (Z.H.L.); mpeqiyi@nus.edu.sg (Y.Q.)

**Keywords:** single-pixel imaging, microelectromechanical systems (MEMS), computational technology, MEMS scanners, MEMS modulators

## Abstract

Single-pixel imaging technology is an attractive technology considering the increasing demand of imagers that can operate in wavelengths where traditional cameras have limited efficiency. Meanwhile, the miniaturization of imaging systems is also desired to build affordable and portable devices for field applications. Therefore, single-pixel imaging systems based on microelectromechanical systems (MEMS) is an effective solution to develop truly miniaturized imagers, owing to their ability to integrate multiple functionalities within a small device. MEMS-based single-pixel imaging systems have mainly been explored in two research directions, namely the encoding-based approach and the scanning-based approach. The scanning method utilizes a variety of MEMS scanners to scan the target scenery and has potential applications in the biological imaging field. The encoding-based system typically employs MEMS modulators and a single-pixel detector to encode the light intensities of the scenery, and the images are constructed by harvesting the power of computational technology. This has the capability to capture non-visible images and 3D images. Thus, this review discusses the two approaches in detail, and their applications are also reviewed to evaluate the efficiency and advantages in various fields.

## 1. Introduction

In conventional imaging systems, the scenery is usually focused by camera lenses onto a focal plane where it is captured by a pixel array detector. Current cameras can obtain an image with millions of pixels using a silicon-based sensor chip, owing to the mature technology of complementary metal-oxide-semiconductor (CMOS) and charge-coupled devices (CCDs). The number of pixels on the sensor chip is usually adopted to evaluate its performance and marketing value, but this type of sensor only works efficiently at the specified wavelength range. It is noticed that there is increasing demand for imaging systems that can operate at wavelengths unavailable for silicon-based sensors to meet different sensing applications, such as far-infrared and deep ultraviolet sensing. Sensor arrays in these operating ranges are either expensive or unavailable; thus, single-pixel imaging (SPI) technology provides an alternative method to construct an imager with just one single-pixel detector in these cases. Based on the general architectures, the SPI systems can be divided into two main research subcategories, namely the scanning-based approach and the encoding-based approach.

The scanning-based method originates from the reported televisor in 1929 [1], which employs a light sensitive detector and a spiral-perforated disc, the Nipkow disk [2], to raster-scan the target scenery at different positions. Modern scanning imagers are typically established with various scanning devices to direct the light from the scenery onto a single-pixel photodetector, and each pixel of the scenery is separately scanned and recorded at each scanning step. This type of imager is commonly applied in imaging where the operating wavelengths cannot be sensed efficiently by silicon-based sensor arrays, where its performance may be affected when the light intensity from each pixel is too weak to be efficiently detected, for example in long-range imaging. The performance of such mechanism is highly related to the mechanical properties of the applied scanners, and its pixel resolution is decided by the achievable number of measurements (scanning points) employing specific scanners. With the encoding-based approach, the scenery is usually encoded with time-varying patterns generated by a spatial light modulator (SLM), while the encoded light is synchronously collected with a single-pixel detector. In this case, the image is recovered through specified algorithms, and thus the efficiency of this method mainly relies on the power of computational technology instead of hardware. The encoding system is generally performing a sampling process to sense the light from multiple pixels at each sampling step, thus benefiting from the improved detected efficiency and low dark count. In addition, some well-established mathematical theories allow us to compress the signal during the acquisition process to reduce sampling time and data storage requirement [3], which is significant in real-time imaging systems [4] or hyperspectral imaging systems [5]. However, the computational overhead is still an important consideration when constructing an encoding-based SPI system.

Over the past few decades, the miniaturization of imagers has drawn the attention and interest of researchers to meet the increasing demand of portable devices for field applications. Considering that microelectromechanical systems (MEMS) technologies are capable of integrating multiple functionalities within small devices, this technology has been explored to develop various compact SPI systems by miniaturizing the scanners and modulators in the two approaches mentioned. Until now, many MEMS scanners have been widely explored in truly miniaturized imaging systems, such as endoscopes. In addition, there also exist many demonstrations of the encoding-based SPI systems using MEMS technology for these imaging areas where traditional cameras get limited efficiency. This paper reviews these single-pixel MEMS imaging systems with the purpose of providing useful information to anyone planning to construct a compact imager for a specific application. The operating principles of the two methods will be briefly introduced in this paper, followed by a review of the typical system architectures. This paper also focuses on the applications of single-pixel MEMS imaging systems to evaluate its efficiency and advantages in various industrial fields. The potential of this technology is also described in this paper considering its latest imaging applications, such as 3D imaging.

## 2. Scanning-Based Approach

**Figure 1:**
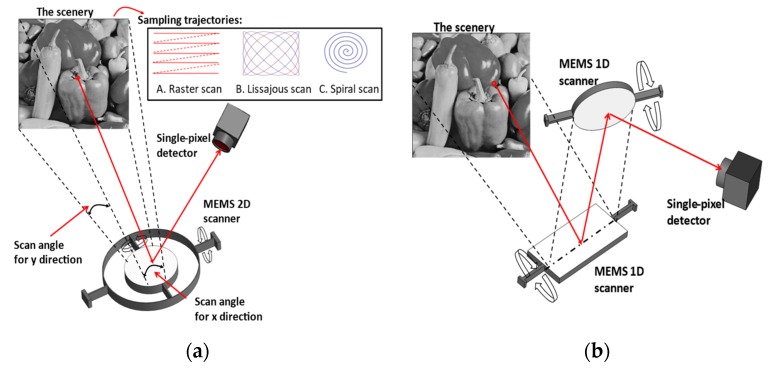
Schematic of two scanning strategies to build a single-pixel imaging system: (**a**) using a single dual-axis scanner; (**b**) using two orthogonal single-axis scanners.

After the first scanning mirror was reported in 1980 [6], MEMS scanners have become an important research topic in the MEMS area and also drive the development of portable and truly miniaturized imaging systems. Compared with traditional scanning optical devices, such as galvanometric scanners, MEMS scanners are preferable in field applications, considering their capability of miniaturization, lower power consumption and high performance at resonant driving conditions. Figure 1 depicts two general approaches to build an SPI system using MEMS scanners. In the first approach, a scanning imager can be implemented by a single dual-axis scanning mirror, which scans the target scenery and directs the scanned light intensity onto a single-pixel detector. In the second approach, two orthogonal single-axis scanning mirrors are employed to scan the scenery instead, thereby increasing the system complexity. When designing such imagers, scan angles and scan speed should be designed properly to ensure the appropriate field-of-view and framerates. In addition, it is also noticed that some scanners have been developed with the ability of additional axial scan to achieve 3D images, which are desired in imaging applications.

The inset of Figure 1a schematically gives the scanning trajectories of three commonly applied strategies in MEMS scanning imagers. Raster scan was the first sampling strategy used for SPI systems and consists of slow scanning and fast scanning in two orthogonal directions. The acquisition time of this imager is mainly determined by the lower scanning frequency, which should be increased to acceptable levels for real-imaging applications. Such a scanning mechanism can be easily obtained with torsional MEMS scanners or fiber scanners, and thus it is widely applied in MEMS imaging systems. Flexible micro-springs are desired in the architecture of torsional scanners for slow motion to ensure the scanning range (field-of-view) [7,8,9], while high driving voltage is required for fiber scanners. Another solution to scan the scenery is to utilize the Lissajous scan, which can conduct simultaneous biaxial high frequency scans. Its scanning range and framerate can be ensured by operating it at resonant mode, and thus this strategy benefits from the mechanical stability and low power consumption [10]. It is noticed that spiral scanning is also employed in some scenarios [11] using a bare-fiber scanner, which allows for miniaturization. To achieve the shown trajectory in the inset, two separate signals with the same frequency are set with a 90° phase-shift to drive the scanner along two orthogonal axes, while the amplitude along the radius is modulated during the scanning process. However, it should be noted that the scanning speed and illumination density distribution condition may affect the performance of this scanning approach [12,13]. One can choose the proper sampling strategy according to the application requirements, and some selection rules can be found in [14].

### 2.1. Microelectromechanical Systems (MEMS) Scanners Technologies

There are four predominant driving mechanisms commonly applied in the construction of MEMS scanners, namely electrostatic, electromagnetic, piezoelectric and electrothermal actuation. Each actuation approach has its potential applications as well as limitations in image capture, and it should be noted that a variety of MEMS scanning mirrors have also been reported in other fields, such as MEMS displays and optical communication. This paper will focus on the application of MEMS scanners to construct SPI systems, and thus much attention is focused on these parameters, which affect the performance of imagers. More specifically, a large field of view is typically achieved with large scan angles, while a higher scan frequency can increase the framerate. It should be noted that mirror smoothness is also an important parameter in imaging applications considering that the deformation of the mirror may distort the images. In this section, a brief introduction is given on the basic working principles of the four actuation mechanisms, followed by some imaging applications of each technology.

#### 2.1.1. Electrostatic Actuation

**Figure 2:**
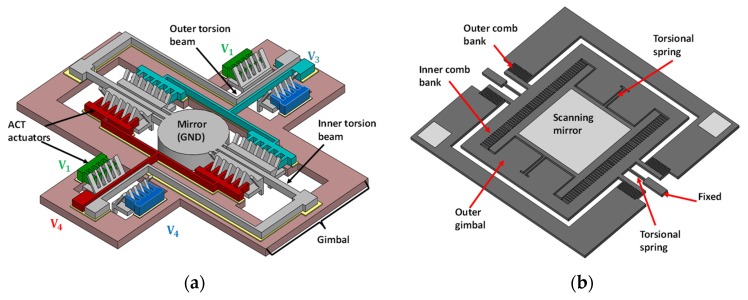
Schematic drawing of (**a**) the microelectromechanical systems (MEMS) scanner using an angular vertical-comb (AVC) actuator [15] and (**b**) the scanning mirror developed on single-crystal silicon and actuated with two vertical-comb structures [17].

Considering the fabrication complexity and the ease of integration, electrostatic actuation is an attractive actuation method for the MEMS scanner. This actuation mechanism utilizes the electrostatic force generated between two oppositely charged components to tilt the micromirrors. There are two architectures, comb-drive actuation and parallel plate actuation, to be explored for the driving of MEMS scanners in imaging applications. In comb-drive architectures, the movable comb structures are typically offset from the fixed comb structures at the initial condition, and the electrostatic force can be generated to move the movable fingers up and down with a supplied voltage. The micromirror connected with the movable comb structures can then be titled to the specified scan angles. Figure 2a shows a MEMS scanner actuated by an angular vertical-comb (AVC) actuator [15]. The device was constructed through surface and bulk micromachining processes and can achieve a scan angle of ±6° under non-resonant operation, while the resonant frequencies were measured to be 463 and 140 Hz for the inner and outer axis, respectively. In addition to providing 2D images, various efforts have also been devoted to building 3D scanning architecture, which can capture the side view as well through specific optical configurations or scanner design. In this case, the micromirror was integrated within an optical coherence tomography (OCT) catheter of 5 mm diameter and can provide a side view with 2 mm working distance by mounting the mirror tilted to the optical path at ±45°. This system can achieve a 1.8 mm × 1 mm × 1.3 mm field of view with 500 × 500 × 1000 pixels for in vivo imaging applications, and the frame rate was reported to be 4 frames/s. In reference [16], a 2D scanner was demonstrated with gimbal-less electrostatic actuators. Separate fabrication processes were conducted to form the actuators and the mirror with a size of 1 mm × 1 mm, followed by manual bonding. The tilt angle was measured to be 20° under resonant conditions, and the resonances were observed to happen at 1.8 and 2.4 kHz for the two axes with the single-crystal silicon actuators. The presented device was integrated in an OCT imaging probe (diameter < 4 mm) to achieve 3D imaging combined with an additional axial scan. The size of the captured volume images was 1 mm × 1 mm × 1.4 mm corresponding to 400 × 200 × 560 pixels, and the 3D OCT imaging successfully captured clear 3D images of tissues with a framerate of 3 frames/s.

Figure 2b shows another example of a 2D scanning mirror developed on single-crystal silicon for two-photon fluorescence imaging [17]. The mirror with a size of 0.75 mm × 0.75 mm was actuated with two comb banks to achieve a 2D scan, and the resultant scan angle was measured to be about 16°. The fast scanning axis can achieve a scanning frequency of up to 3.5 kHz, and later a similar structure was reported for the construction of a compact dual-axis confocal microscope with a framerate of 8 frames/s. The resonant frequencies were measured to be 500 Hz for the outer axis and 2.9 kHz for the inner axis. The scan angles for these two axes are reported to be ±6.2° and ±3.6°, respectively [9]. Various MEMS scanners have been widely reported in other imaging architectures using this actuation principle, including the OCT [18] and confocal microscopy or endoscop [19,20,21,22].

**Figure 3:**
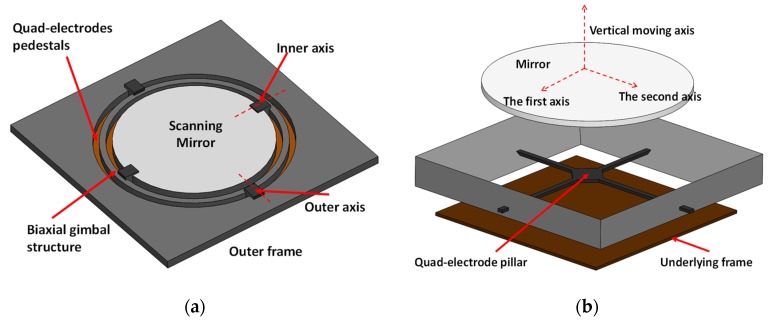
Mechanical structures of (**a**) the MEMS scanner using quad-electrodes to achieve a 2D scan with a gold-coated mirror [26] and (**b**) the quad-electrode pillar utilized to drive the mirror combined with electrostatic actuation between the mirror and bottom basement for 3D imaging [30].

Another scheme is to utilize a fixed electrode to attract the movable micromirror platform to generate the scan motion. In the field of imaging, this type of actuation was firstly adopted to develop a single axis scanner in a confocal microscope where two such scanners were used to achieve a functional 2D scan [23], as described in Figure 1a. Two dimensional scanners can be achieved with quad-electrodes, and these scanners have been reported in miniaturized configurations for confocal microscopy [24] and OCT systems [25]. Figure 3a shows a 2D scanner for OCT imaging application [26], from which it can be seen that the central mirror is connected to the basement through a biaxial gimbal structure, while the quad-electrode pedestals are implemented on the bottom of the mirror with a gap at the static condition. This structure obtained a 2D scan with an achievable angle of ±4° with a 130 V DC power supply, and this device could be operated at its resonant frequency to achieve the same scan angle with 20 V AC plus 60 V DC bias voltage. Compared with the comb-drive actuation, this type of actuation method typically results in a non-linear relationship between the deflection angle and the supplied voltage when DC voltage is used. The resonances of the inner and outer axes were observed to happen at 472 Hz and 399 Hz, respectively. At resonant operation, a linear relationship was obtained between the scan angle and the supplied AC voltage.

It is worth mentioning that such an actuation principle can be extended to develop focus-adjustable MEMS micromirrors by using the attraction force between the mirror and the bottom frame. Such micromirrors have been applied in various OCT imaging systems [27,28,29], and functional 3D scans can be achieved by integrating this architecture with a quad-electrode based 2D scanner, shown in [30,31]. These architectures are quite similar to the scanner shown in Figure 3a, despite the slightly different mirror geometries and fabrication processes. A quad-electrode pillar was constructed separately and manually inserted to the cavity between the mirror and the underlying frame to achieve a 2D scan, as shown in Figure 3b. Electrostatic attraction is generated between the mirror and its underlying silicon frame to change the focus of the micromirrors. The device [30] was reported to have a scan angle of around ±5° under a 140 V DC voltage supply, while a ±4° scan angle was observed under the resonant frequency at around 216 Hz with 40 V AC voltage. With additional electrostatic actuation, the focus can be adjusted from infinity to 50 mm with less than 100 V. This system was demonstrated for endoscopic applications. In various imaging architectures, this method can provide a framerate of more than 10 frames/s with the resolution of few microns.

#### 2.1.2. Electromagnetic Actuation

**Figure 4:**
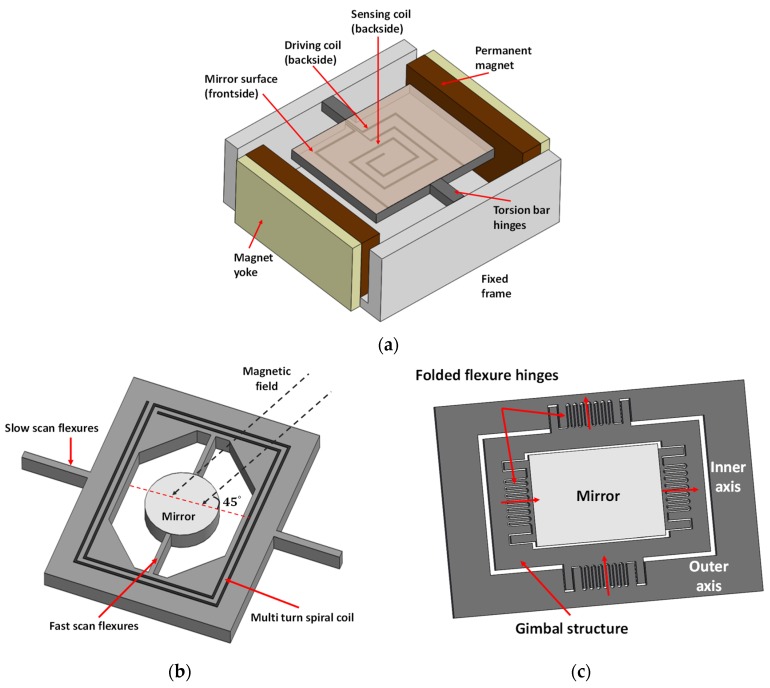
Schematic of the mechanical structures of a (**a**) 1D scanner in Olympus confocal microscope [32]; (**b**) 2D scanner with an external magnet set at 45° to the plane of the scanning axis [34]; (**c**) 2D scanner driven by the glued magnets on the side of the mirror [35].

Electromagnetic actuation is another actuation principle, which utilizes magnetic torque to tilt the microscanner. Two actuation schemes, moving coils and moving magnets, have been applied to develop MEMS scanners. Figure 4a shows an example of moving coils used in a commercial Olympus confocal microscope [32]. External permanent magnets were employed to drive the micromirror on which the driving coils were implemented through electro-plating. The mirror (4.2 mm × 3.0 mm) was constructed on a wafer with 300 μm thickness to maintain the mirror flatness, thus ensuring the imaging quality. The scanner was designed with a resonant frequency of roughly 4 kHz to achieve a fast scan, with its scan angle reaching 8°. It should be noted that the reported scanner was only designed to allow a single-axis scan, and single dual-axis scanners have also been explored for optical coherence tomography, such as the non-resonant scanners shown in [33]. Figure 4b gives the schematic of a single dual-axis scanner using the moving coils scheme, reported in [34], for imaging and display applications. A functional 2D scan was achieved by using mechanical coupling between the mirror (diameter: 1.5 mm) and the outer frame structure. Multi-turn spiral driving coils were implemented within the outer frame through typical bulk MEMS fabrication techniques. An external magnet was set at 45° to the plane of the scanning axis, and thus the outer frame was driven in two orthogonal directions. Such architecture can get full scan angles of 65° and 53°, respectively, and the scan frequency reached up to 20.5 kHz for the fast scan and 60 Hz for the slow scan, respectively. Similar structures can be found in confocal microscopy reported in [35], and Figure 4c shows a two-axis scanning mirror with a thin magnetic layer glued on the backside of the mirror. The depicted mirror is used to construct a MEMS catheter 2.8 mm in diameter for 3D endoscopic OCT and can obtain a scanning range of ±20° in both directions. Additionally, it can be operated at resonant frequencies of 450 Hz and 350 Hz for the depicted inner axis and outer axis, respectively. It is worth mentioning that the required operating voltage is relatively low using this actuation method and thus benefits its applications. Apart from these silicon mirror structures, other types of MEMS scanners have also been explored for imaging applications using this actuation method, such as polydimethylsiloxane-based mirrors [36] and microlenses [37].

#### 2.1.3. Piezoelectric Actuation

**Figure 5:**
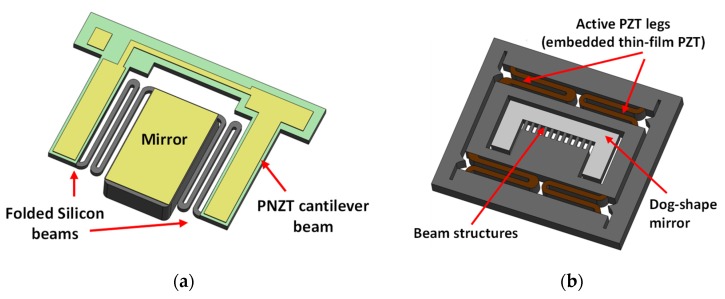
Mechanical structures of (**a**) the MEMS scanners indirectly actuated by a 13%-Nb-doped PZT (PNZT) cantilever beam [40]; (**b**) the MEMS scanners actuated by active lead zirconate titanate (PZT) legs to achieve the vertical translation and rotated by the mechanical couple into the gimbal platform [41].

Piezoelectric actuation provides another solution to develop functional MEMS scanners by incorporating the piezoelectric effect of specific materials, such as lead zirconate titanate (PZT), after PZT was reported to be micromachinable in MEMS devices [38]. This actuation principle typically provides large static deflection and high driving force, and it also allows acceptable resonant frequencies without consuming much power. The original scanner structures were established by incorporating the PZT thin film into a cantilever beam where the mirror was implemented on the topside. Thus, when a voltage was supplied to one side of the PZT, the thin film would expand in a specified direction to generate the driving force, and the whole beam could be titled to obtain a scan angle. In these architectures, there exists a trade-off between actuation angles and the mirror flatness. A fabrication procedure, reported in [39], was designed to independently fabricate the mirror and cantilever beam with defined thickness on a thermal silicon dioxide wafer through a two-step etching and releasing process. Thus, this method could maintain the mirror flatness with a scan angle of ±7° and ±8° at static and resonant conditions, respectively. In this case, the resonant frequency is affected by the length of the beam. More specifically, a shorter beam can result in a higher resonant frequency and reduced scan angle. This PZT cantilever beam structure was employed in a Fourier-domain OCT system to demonstrate its imaging capability. Different from the unit structure, Figure 5a gives another 1D optical scanner where the mirror is connected with two piezoelectric cantilever beams through the folded silicon beams, and thus the scanner is indirectly driven by the PZT beams to ensure the mirror flatness [40]. A so-called 13%-Nb-doped PZT (PNZT) film was employed to actuate such scanners, which shows the potential of achieving large scan angles with piezoelectric actuation by optimizing the properties of applied PZT materials. The presented device, 3.4 mm × 2.5 mm in size, can achieve a scan angle of 152° under a resonant frequency of 394 Hz, and the scanner was successfully demonstrated in a swept-source OCT system. 

In addition, a larger scan angle can be achieved by employing a thicker PZT film, and Figure 5b indicates an architecture using a thin PZT film to drive the scanner to rotate and which is suitable for cross-sectional imaging in a dual-axis confocal system [41]. It can be seen that a thin-film PZT was implemented within the outer legs to actuate a gimbal platform in the vertical direction. Specific beam structures were designed to connect the gimbal platform with the mirror, which was designed as dog-bone shape. The inner rotational scanning mirror was actuated through the coupled mechanical motion from the gimbal platform, and the whole device was excited at its resonant frequency. Even though the scanning mirror only resulted in ±5.5° scan angle with 2V AC driving condition, 200 μm displacement could be achieved in the vertical directions owing to the usage of thin-film PZT under 18 V. The resonant frequency of the device was measured at 2.8 kHz and could be increased further by etching holes on the mirror platform. The device consumed power on the order of 30 μW; 2D MEMS scanners using this actuation mechanism can be achieved by modifying the beam structures embedded with the PZT thin film [42,43]. It should be noted that a more complicated fabrication process is required to develop the scanners using piezoelectric actuation, owing to the additional embedded PZT film. In imaging applications, this actuation method can achieve a high framerate with several tens of frames/s, but the resolution may be degraded with high scanning speed in this case.

#### 2.1.4. Electrothermal Actuation

**Figure 6:**
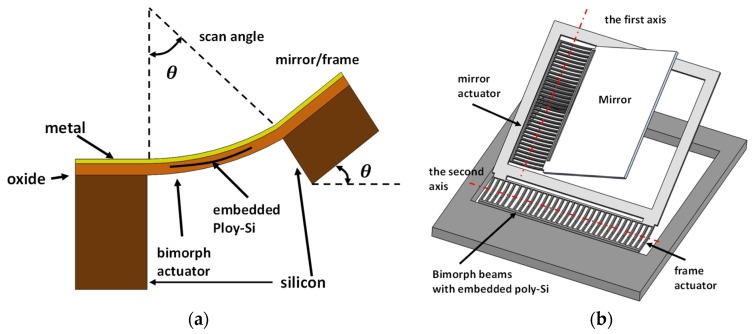
Schematics of (**a**) the working principles of bimorph structures [44]; (**b**) the 2D MEMS scanner using two axial bimorph beams to drive the frame and the mirror [45].

Scanners using electrothermal actuation are generally constructed with thin bimorph structures whose constituent materials have contrasting thermal expansion coefficients. Inspired by the original architecture of thermally actuated micromirrors [44], this type of MEMS scanner was first demonstrated for endoscopic OCT imaging applications with a multilayer Al/silicon oxide mesh structure. The working principles are schematically shown in Figure 6a, where a current was supplied to a polysilicon heater embedded within a beam with mesh structure in this case, and the mesh structure was bent down, and the mesh structure was employed to connect one edge of the mirror and the substrate. Thus, the single-axis scan motion could be realized to get a rotation angle of 17°, and the resonant frequency was observed to be 165 Hz. The reported system consumed 15 mA DC and had an imaging resolution of 20 μm. Later, a dual axis scanner was reported with similar structures for the same application, as shown in Figure 6b [45]. The mirror was connected to the outer frame with electrothermal bimorph beams, while the same beam structure was used to connect the frame to the substrate. Thus, this presented device could be actuated along two orthogonal axes, and the achievable scan angle was up to 40° with the supplied 6.3 mA AC. The resonance of the mirror was measured at 445 Hz and 259 Hz for two separate scanning axes. 

**Figure 7:**
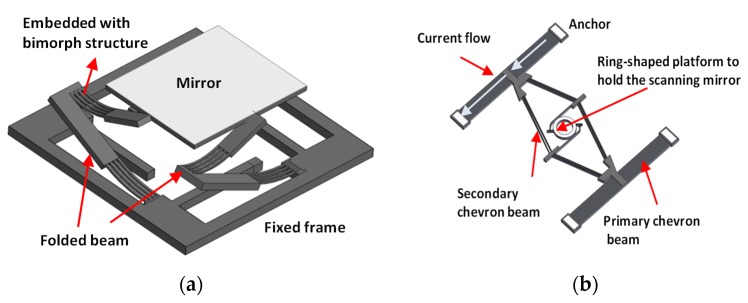
Mechanical structures of (**a**) the 2D scanners with bimorph beam structures to ensure the rotation along the central axes of the mirror [46]; (**b**) the circumferential scanner actuated by cascaded chevron beam to rotate the center ring-shaped platform for the scanning mirror [48].

It should be noted that the rotating action along one edge of the mirror may shift the optical path during the scanning process, thus increasing the burden on the optical design for real imaging application. Thus, an alternative architecture was reported to ensure the mirror can rotate along the central axis by modifying the bimorph structures, as shown in Figure 7a [46]. A folded beam with embedded bimorph structure was employed to support and connect the mirror at the central points of its corresponding edge. The micromirror resulted in a ±15° scan angle under a resonant frequency of 400 Hz, and driving condition was set at 5.5V and 15 mA. This micromirror was successfully integrated into a probe (outer diameter: 5.8 mm) for OCT imaging, and the probe size could be further reduced to 2.8 mm using a hidden actuator [47]. Different from fabricating the mirror and actuators as a unit, Figure 7b shows an actuator that utilizes cascaded thermally actuated chevron beams to rotate the central platform with a mounted micro-pyramidal polygon reflector [48]. When current flows through the chevron beams, the beam moves forward owing to the thermal expansion effect, and thus the central platform is driven to rotate. This cascaded structure can achieve a larger rotational angle at 41° compared with the single beam design, and this device was designed to perform circumferential scanning with a 328° scan angle. This device was demonstrated for OCT imaging. Other thermal actuated scanners have also been developed in OCT imaging systems [49] and multiphoton imaging [50] and confocal microscopy [51]. However, the power consumption is still a challenge for this actuation method.

### 2.2. Applications of Scanning-Based Single-Pixel Imaging System

The scan-based method typically fits well with the SPI technology, since only one detector is required in these cases to collect the light from the applied MEMS scanners. In these systems, the imaging performance and quality is mainly determined by the applied MEMS scanners. Various MEMS scanners structures were reviewed in the previous sections with the purpose of indicating the performance of such scan-based methods, which are typically dependent on the efficiency of MEMS scanners. With MEMS scanners providing efficient 1D, 2D or 3D scanning, various imaging systems can be achieved with related optical configurations. Thus, the MEMS scanner may be broadened for various applications with proper optical system design. Until now, biological imaging has seen the major application of imaging systems using MEMS scanners, mainly including OCT, confocal microscopy and multiphoton microscopy.

**Figure 8:**
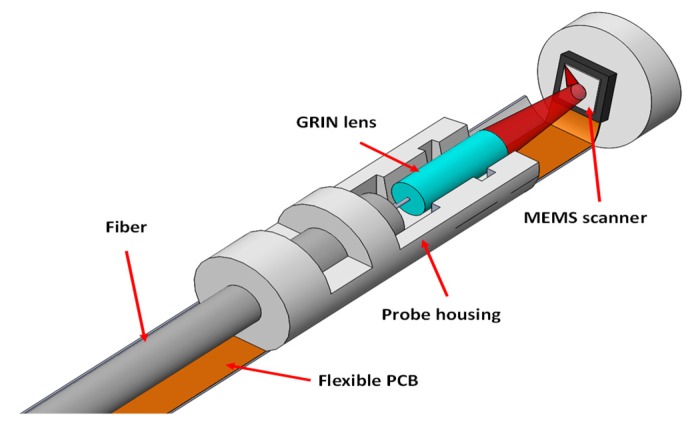
Schematic of a compact optical coherence tomography (OCT) probe (diameter: 2.8mm) with thermoelectric-actuated scanner, hidden actuators, a graded-index (GRIN) lens, a flexible printed circuit board (PCB) and a probe housing made from a fluorinated ethylene [47].

Optical coherence tomography (OCT) is a technology capable of providing a fine resolution with micro-scale; thus, this technology has much significance in disease detection, such as cancer. This system constructs optical scattering image with low-coherence interferometry, and the SPI technology supports the flexibility of the applied light source, which may affect the resolution. By integrating MEMS technology, this system can be miniaturized into a small imaging probe for field applications [52,53,54,55,56,57]. Figure 8 shows a compact design of an integrated OCT imaging probe constructed with the electrothermal actuators [47]. With hidden actuators, the dimeter of the presented probe is only 2.8 mm, and all required components are integrated to perform OCT scanning. As reviewed, all four predominant actuation methods get their related architectures for OCT imaging, and tunable focal length scanners have also been demonstrated to achieve more functional scanning architectures. More recently, a miniaturized compact OCT catheter was integrated with an array of MEMS scanners based on electrothermal actuation [58]. Adjustable focal length can be implemented with each scanner, which achieves a scan angle at 45°, and thus the reported structure can be adjustable for uneven surfaces, and this catheter was experimentally demonstrated to show its potential in in vivo imaging applications.

**Figure 9:**
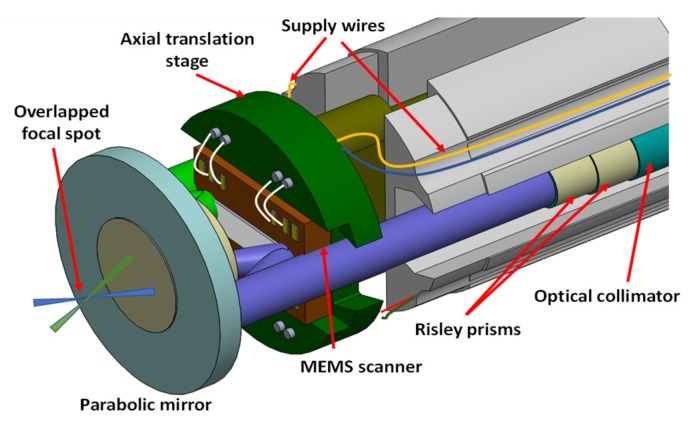
Schematic of a dual-axis confocal microscope with a MEMS scanning mirror and a parabolic mirror to focus two incoming collimated beams [59].

Confocal microscopy is another field where MEMS scanners have been widely explored for several decades, as this technology can provide a subcellular resolution. The exploration of MEMS-based confocal microscopy makes it possible to build portable devices for field testing, considering commercial confocal microscopy is usually limited in lab testing. Figure 9 shows a compact design of confocal microscopy [59], where a single-axis MEMS mirror was adopted to perform scanning. Considering the dynamic range and axial resolution, dual-axis scanning is preferable and also can reduce the complexity of optical systems. In this field, 2D scanners are mainly constructed with electrostatic actuated scanners [60,61,62,63,64], and the scanner, reported in [65], can be switched between the lateral and vertical scanning conditions to support the flexibly of MEMS imaging systems. As we reviewed, the piezoelectric actuation was reported to potentially realize 3D scanning modes [66,67], but the fabrication complexity is still an important consideration to build such PZT-actuated scanners.

**Figure 10:**
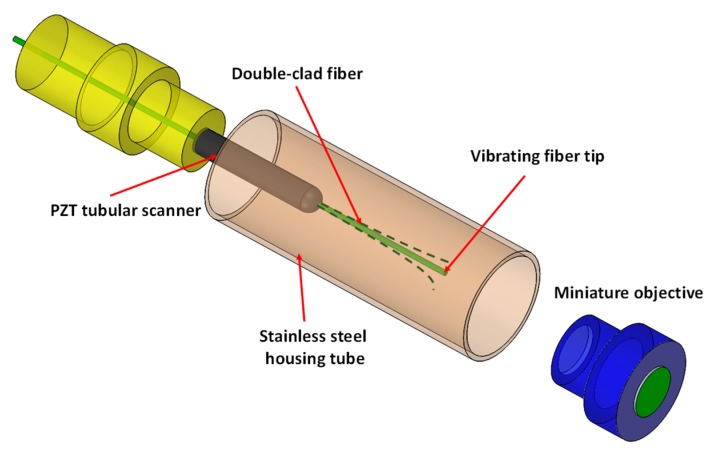
Schematic of the assembly of a fiber scanning system for a two-photon microscope with double-clad fiber actuated by a PZT tubular scanner [70].

Multiphoton imaging, using non-linear light-tissue interactions to provide in vivo images, also benefits from the MEMS technology. SPI technology shows its advantages in this case, since an efficient detector can be used to detect the applied photons in the near-infrared region. All the mentioned driving mechanisms get their related research work in this application field, and the major architectures are constructed with electrostatic actuators [68] and piezoelectric actuators [69]. In this condition, it is worth mentioning another strategy to build a scanning-based SPI system, which is to utilize a vibrating fiber. One example of the fiber scan system is shown in Figure 10 [70]. Unlike traditional scanning architecture, the presented fiber scan system employs a flexible double cladding fiber to realize the laser excitation and emission. The fiber was driven to be vibrated by the PZT tubular scanner. In addition, a miniaturized objective designed with a focal shift along the longitudinal axis was also employed for two-photon microendoscopy. This mechanism has been widely explored by using different actuation methods for various applications, including OCT [71,72,73,74,75], confocal microscopy [76] and multiphoton imaging [77,78,79]. However, it should be noted this method cannot provide flexible scanning trajectories.

It is worth mentioning that MEMS scanners support the flexibility to be employed in various applications through specific optical configurations. Other compact imaging configurations can be found in photoacoustic microendoscopes [80,81,82,83,84], based on the MEMS scanning single-pixel imaging system, to visualize the optical absorption. The scan-based method is also employed in other imaging applications, such as thermal imaging. However, considering the low intensity due to the absorption and scattering, specified algorithms should be implemented in the system to enhance the signal-to-noise ratio [85]. In this case, the potential of computational technology has been shown, which will be discussed in the following section.

## 3. Encoding-Based Single-Pixel Imaging Systems

Different from the scanning architectures, the encoding-based systems are typically constructed with an SLM to encode the scenery with time-varying patterns. The correlated light intensities are then recorded with a single-pixel photodetector. The light intensities from multiple pixels are collected at each sampling step, and thus the overall detected signal can be increased to make such system viable in these cases where the light intensity from the scenery is relatively low, for example in long-range imaging. In addition, this method is beneficial from the computational technology to allow us to reconstruct an image with a compressive sampling strategy, hence greatly boosting the sampling speed compared with traditional scanning methods. Over the past decades, various compact imaging systems have been demonstrated using this encoding method, due to the availability of commercially miniature devices that can be used to generate time-varying patterns. Despite the advantages, it is noticed that the performance of such imaging systems is limited by the properties of the applied detector, including its dynamic range and associated quantization electronics [3]. Besides, the computational overhead is still an important consideration for anyone planning to construct the encoding based single-pixel imaging system.

### 3.1. The System Architecture

The fundamental components of an encoding-based imaging system are the SLM to generate time-varying patterns and a photodetector to synchronously collect the encoded light. The performance of such imaging systems is typically decided by the combination of the applied detector and the SLM. Since this paper focuses on these single-pixel imaging systems using MEMS technology, it is noticed that there are two main types of MEMS modulators that have mainly been explored in single-pixel imaging systems, namely digital micromirror devices (DMDs) and liquid crystals on silicon (LCOS), respectively. The LCOS devices utilize the CMOS technology to develop traditional liquid crystals devices (LCD) on a chip, so this paced device can control the amplitude and phase of the incoming light by employing the special light-modulating properties of LCD. The DMD consists of closely packed mirrors, and each mirror can be tilted to two different directions with respect to the direction of the incoming light. One direction can be integrated with the collection system to collect the overall intensity from the mirrors, which are tuned to this direction (on state = 1), while the reflected light from the other direction will not be recorded (off-state = 0). Thus, binary encoding patterns can be conveniently generated on this type of modulator. There are other types of SLMs that can be used to establish a single-pixel imaging system, and one can select the proper modulators in different regions of interest. A summarized table (Table 1) is thus given to compare these modulators, such as DMD [86,87], LCD or LCOS [88,89], mechanical mask [90,91], customized diffuser [92,93], light-emitting diode (LED) arrays [94,95] and optical phase arrays (OPA) [96,97].

From the given table, it can be seen that the LCOS and DMD potentially satisfy the continuously increasing trend of miniature imaging systems due to their compactness. For instance, each side width of the micromirror is almost equal to a tenth of the width of a human hair, and the width of liquid crystals can reach around 6 μm. In an encoding-based SPI system, the resolution of recovered images is typically determined by the spatial resolution of the applied modulators. The LCOS and DMD provide flexible spatial resolution by combining neighboring element [98] as an efficient pixel, compared with some modulators with fixed pixel resolution, such as a diffuser. However, the LCD/LCOS devices usually require longer modulation time to switch different patterns, while DMDs can be operated at a modulation rate in excess of 20 kHz. Besides, recent works indicate that the OPA and LED devices achieve a modulation rate higher than 1 MHz using fast-switching photonics components to meet the requirement of high framerate. It should be noted that a narrow wavelength range usually limits the applications of OPA and LED, and the properties of applied materials determine the operating range of these imaging systems constructed with mechanical masks and a diffuser. Various mechanical masks have also been demonstrated to perform imaging reconstruction, such as coded apertures, but it should be noted that the encoding patterns are fixed and not programmable after the mechanical mask has been fabricated. Even though DMD can only provide simple modulation patterns compared with diffuser and LCOS, DMD is still the most commonly applied element in current imaging systems, due to its availability and relatively wide range. Therefore, this paper reviews the single-pixel imaging systems using DMD.

**Figure 11:**
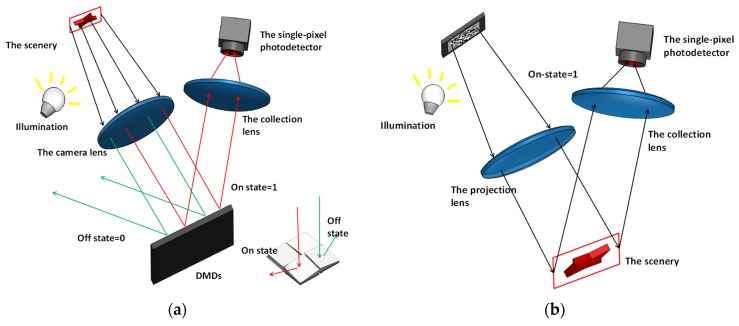
General architectures of two single-pixel imaging systems using digital micromirror devices (DMD): (**a**) the single-pixel camera architecture where the scenery is modulated at the focal plane with inset showing the DMD structure; (**b**) the computational ghost imaging architecture where the scenery is illuminated with the modulated light.

When reviewing the architectures of the encoding-based SPI systems, it is necessary to consider two similar research communities, namely single-pixel camera and computational ghost imaging [99,100]. Figure 11 gives the general description of these two types of systems using DMDs, from which it can be seen that the single-pixel camera typically sets the SLM (DMDs) at the focal plane of the applied camera lens to modulate the light intensities from the scenery. The encoded light is collected with a single-pixel detector, and this architecture is often referred as focal plane modulation. In the architecture of ghost imaging, on the other hand, SLM (DMDs) is employed to modulate the light from the light source instead, and the photodetector collects the reflected light from the scenery after the scenery is illuminated with special patterns. This is also known as structured light illumination. The two types of systems are essentially the same in terms of optical set-up, but it should be noted that some modulators can only be applied in ghost imaging systems where active illumination is required, such as LED arrays. The inset of Figure 11a schematically shows the structure of DMD, from which it can be seen that binary encoding patterns are generated on the DMD by directing the incoming light to two separate directions.

### 3.2. Single-Pixel Detector Technologies

SPI technology benefits from the development of single-pixel detectors, which have high flexibility in working wavelengths and system configuration. The single-pixel detectors are built with various photoelectric effects to convert the incident light into electrical signals through different materials and structures, and photodiode technology is the most commonly employed detection device in SPI systems [101]. Photodiode technology is capable of a large wavelength spectrum by using a wide range of semiconductor materials, such as Ge, GaP, InAs, InSb, InGaAs and so on, compared with the CMOS and CCD detector array whose operating wavelength range is limited by the applied silicon material. For infrared imaging applications, InGaAs photodiodes provide a reliable method to perform sensing tasks for the range between 0.5 μm and 2.6 μm. The applied materials also affect the resulting gains and response time of different photodiodes, and silicon-based photodiodes usually get high response time and high gains. One can properly select the photodiodes with specific semiconductor materials to meet different applications. It should be noted that the dark current of single-pixel detectors is an important consideration for single-pixel imaging systems, considering that the resulting noise may affect the quality of reconstructed images, especially for the encoding-based approach. Therefore, the operating mode that can result in smaller dark current is desired in this application field, such as photovoltaic mode. Otherwise, proper cooling methods should be applied to reduce the dark current. For example, the photomultiplier tube is an efficient type of single-pixel detector technology, which can achieve low dark current rates with suitable cooling systems, but this technology requires vacuum tube technology despite the provided larger sensitive area [102]. Compared with traditional CCD and CMOS sensor arrays, single-pixel technology also allows the usage of detection a device with high sensitivity to conduct low-level-light detection, such as avalanche photodiode (APD) [103]. In addition, single-pixel detector technologies have also widely been explored to provide single-photon sensitivity, such as the superconducting tunnel junction based detector and the quantum-dot field-effect transistor based detector [104], so the resulted imaging systems are able to realize long-distance imaging tasks that cannot be done in traditional cameras.

Therefore, various detector technologies support the flexibility of SPI systems to work at any wavelengths by using suitable detection devices. In addition, the development of cutting-edge detectors is a popular research direction, so SPI technology also benefits from these newly developed detectors since they are usually demonstrated in the single-pixel form. Furthermore, it is always a challenging task to integrate multiple pixels of a newly constructed detector into an array, especially when non-silicon technology is involved. Therefore, the fabrication of such a detector array will be costly, making this method less attractive in field applications, for example in infrared imaging. It should be noted that traditional cameras are still dominant in the visible range considering cheap detector arrays are available with high pixel resolution up to several megapixels. However, detector arrays are typically expensive or even unavailable for some wavelengths, and thus SPI technology provides a competitive edge over traditional detector array based imagers in terms of detector costs in these cases. For example, an InGaAs IR camera generally costs several thousand dollars, compared with a photodiode, which costs about 100 dollars with satisfactory detection performance. 

In addition, the cost of MEMS-based SPI systems also comprises the applied MEMS elements, including the MEMS scanners and modulators. Commercial MEMS scanners using electrostatic actuation cost around 1000 dollars, which still shows the cost advantage over traditional detector arrays. It should be noted that the cost of MEMS scanners may increase with more complicated fabrication processes, for example with piezoelectric actuation based scanners. The scanning-based approach gets its unique applications in biological imaging fields where detector arrays have limited efficiency. For the encoding-based approach, the additional cost comes from the MEMS modulators (DMD), which has been well-established with high pixel resolution and affordable cost. Considering various demonstrations of SPI systems, the average cost of encoding-based systems can be estimated to be around 1000 dollars per megapixel [105], which is generally several times less than that of detector arrays in non-visible ranges. In addition, the cost of such SPI imagers can be maintained over a large wavelength range, compared with the increasing cost of a detector-array camera in the non-visible range. Despite the mentioned advantages of SPI technology, the single-pixel detection element typically requires more sampling time to sequentially record the scanned or encoded light intensities, while traditional cameras capture images in one shot. That is the main reason why MEMS scanners and modulators with higher operating rates are desired in this case to speed up the acquisition. Besides, the encoding-based approach can take advantage of computational technology to reduce sampling time.

### 3.3. The Mathematical Interpolation and Sampling Strategies

In encoding-based SPI systems, the target image can be considered as a two-dimensional matrix, and each element in the matrix represents the light intensity information at its corresponding location. This matrix can be expanded to a vector ***X*** with *N* unknown light intensities, and the time-varying patterns can be expressed by a matrix Φ with dimension M×N. Each row of the encoding matrix is the patterns Φ(j) generated by DMD at each sampling step, and thus the detector records the correlated light intensities between the encoding pattern and the target image, which can be mathematically expressed by the inner products of Φ(j) with ***X***. After a complete sampling cycle, *M* measurements can be obtained to form the measurement vector ***S*** as follows:

***S*** = ***ΦX*** (1)

A complete orthogonal matrix was first employed to construct an encoding-based SPI system, such as Hadamard basis and Fourier basis [106,107,108]. Despite the fact that this method can reduce the sampling noise [109], this type of system still requires *N* measurements to reconstruct an *N*-dimensional image. When the number of measurements is smaller than the signal’s dimension (*M < N*), two other types of sampling strategies can be employed to boost the acquisition process, namely compressive sensing and sub-orthogonal sampling, respectively. The sub-orthogonal sampling utilizes a sub-basis Φ (*M < N*) drawn from an orthogonal matrix to compress the signal during the sampling process. The basis is required to be incoherent with the spatial properties of the target images, thus making it possible to reconstruct it through specified algorithms. Compressive sensing (CS) [110] indicates a signal can be accurately recovered with high-probability with much fewer measurements by using its sparsity in some transform bases, after the signal is modulated with pseudo-random patterns.

The efficiency of CS depends on the assumption that the signal ***X*** tends to be sparse in a known basis ***B***, such as JPEG algorithms. Thus, the sampling process can be modelled as follows after representing the target image with a basis B and sparse vector θ:

***S*** = ***ΦBθ*** (2)

where the θ is usually called a *K*-sparse vector, which means only *K* elements in the vector are non-zeros. The sparse vector can be efficiently reconstructed through $l_{1}$ minimization algorithms with an adequate number of measurements (*M*), and there are two types of approaches to solve this optimization problem, including the convex optimization algorithms or greedy algorithms. However, it has been proved that pure $l_{1}$ minimization algorithms are vulnerable to noise, and thus a more general approach is usually applied to increase its robustness by introducing an additional $l_{2}$ minimization term:

(3) $$minimize\;{\left\|X\right\|}_{1}\cdot subject\cdot to\cdot {\left\|S-\Phi B\theta \right\|}_{2}\leq \delta$$

where δ is the acceptable noise boundary in the real applications. The reconstructed accuracy is ensured by minimizing the $l_{2}$ norm function of the measured signals and the signals resulting from the predicted signals, and the $l_{1}$ norm guarantees the sparse solution to satisfy the mentioned assumption. Many other types of algorithms can be found in the literature [111,112,113] to solve such problems, but it can be seen that most algorithms are still based on the $l_{1}$ and $l_{2}$ minimization problem. It is worth mentioning that an image can be reconstructed by exploring its sparsity in the pixel domain using the minimization of the total variation (TV) or the total curvature (TC) of the images [114,115]. It is worth highlighting that the time required to capture in the encoding systems consists of the sampling time and the data processing time. Even though the usage of CS allows us to speed up the sampling process of a single-pixel imaging system, the described algorithms significantly increase the computational overhead to limit its real-time applications. Typically, this method has been successfully demonstrated in these applications, which allow the post-processing [116,117].

Generally, the single-pixel imaging systems are constructed with three main sampling strategies mentioned above, and a summary of these strategies is given in Table 2. Even though the complete orthogonal sampling could not provide the competitive edges over traditional scanning methods in terms of sampling time, this sampling strategy has wide applications considering its capability of reducing the sources of noise. Additionally, the Hadamard transform sees the major application of DMD to construct a single-pixel imager. Recently a three-step phase-shifting encoding pattern was efficiently generated on DMD to achieve a functional Fourier-transform, despite the fact that the required number of measurements was increased 1.5 times [4]. The efficiency of the sub-orthogonal sampling strategy is dependent on the prior knowledge of the spatial properties of the target scenery, which is required to be incoherent with the chosen sub-basis, and fast reconstruction algorithms can be incorporated with this strategy to significantly boost the reconstruction time. Many imaging architectures have been reported to obtain the prior knowledge in [118,119,120,121,122] before performing the sub-orthogonal sampling. For example, a co-aligned traditional camera was used to get a stream of 2D images continuously [119], and the scenery was sampled with low resolution to get some prediction of the images for later high resolution reconstruction [120]. To optimize the reconstructed results, deep-learning has also been explored to predict the prior information [121]. CS theory has been explored widely in 2D images due to the fact that the natural 2D images tend to be sparse in some domains, especially for visible bandwidths [3,123], but the computational overhead should not be neglected. Additionally, one can select the proper sampling strategy according to the requirements of sampling speed, achievable prior knowledge and reconstruction time.

### 3.4. Applications of Encoding-Based Single-Pixel Imaging Systems

Compared with the scanning-based SPI systems, the encoding method has wide applications in various fields, and the demonstration for the visible range can be easily found in previously reviewed architectures. However, considering that current cameras efficiently work at the visible range with satisfactory pixel resolution and low computational overhead, in this section we mainly focus on the applications of the MEMS encoding imager in these scenarios where the traditional cameras have limited efficiency, or the detector array based imagers are costly.

#### 3.4.1. One Dimensional Imaging: Spectrometer

**Figure 12:**
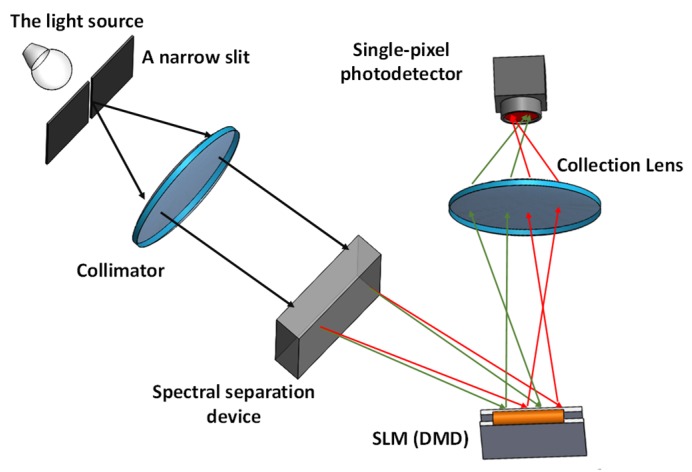
A typical architecture for building a spectrometer using DMD.

Spectral acquisition, commonly conducted with a Fourier-transform spectrometer, plays an important role in various fields, including chemical and biological analysis. However, the FTIR spectrometer is limited to the laboratory testing, owing to its large size and fragile moving optical components. Thus, miniaturized spectrometers have been explored using DMD, owing to the flexibility and advantages of DMD. Figure 12 indicates a typical architecture of the spectrometric system based on DMD, from which it can be seen that a narrow slit is typically employed to select the light into the system, and the incoming light passes through a spectral separation device, such as a prism or grating. The dispersed spectrum is encoded by the encoding patterns generated on the DMD. Finally, a single-pixel detector is used to collect the light from the DMD. The DMD based spectrometer was firstly demonstrated in visible ranges [124], and later this type of spectrometric system began to be reported in non-visible wavelengths. Until now, the DMD has been applied in various fields, including analytical atomic spectrometry [125], chemical sensing [126] and infrared sensing [127]. Besides, CS theory was also utilized to reduce the required number of measurements, thus significantly reducing the sampling time [128,129]. It should be noted that the real resolution of a spectrometric system is also dependent on the optical set-up, so some improvement and analysis have been done by modifying the mask pattern widths to improve the performance of the DMD-based spectrometers in near-infrared sensing applications [130].

**Figure 13:**
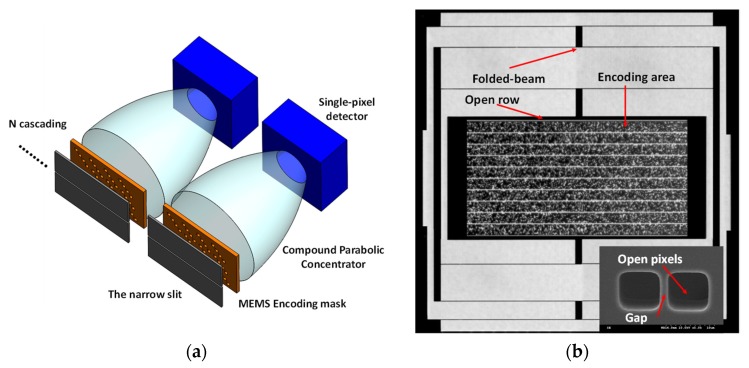
(**a**) The schematic drawing of a MEMS vibrating mask with cascaded structures, with compound parabolic concentrator glued on single-pixel detector [132]; (**b**) The microscopic picture of the compressive sensing (CS) vibrating mask with the inset showing the SEM image of the pixel structures [133].

More recent explorations have been reported by using the MEMS mechanical mask consisting of sequential encoding rows [131]. Each encoding row contains open pixels and closed pixels, which were fabricated through the typical MEMS fabrication process. The light can pass through the open pixels and be recorded with the applied single-pixel detector, thus representing 1 in the encoding matrix. Thus, binary encoding could be implemented through this structure, and the MEMS mask was driven to scan across a fixed narrow slit, which was just placed in front of the MEMS mask. In this way, time-varying patterns were generated along the slit to encode the dispersed spectrum. This encoding method was firstly demonstrated with the Hadamard transform using an electromagnet actuator. Even though the mask was designed to be operated at its resonant frequency, the travel range was still limited to restrict the number of encoding row to be implemented, thus affecting the achievable pixel resolution. To overcome the limitation, a new mask structure was reported, as shown in Figure 13a [132]. In this design, multiple encoding masks could be cascaded along the slit direction, and each mask was implemented with the Hadamard matrix. This method can potentially increase the dimension of the reconstructed signal by *N* times with *N* cascaded MEMS elements, due to the fact that each MEMS mask can achieve a complete orthogonal sampling with a given MEMS travel range. In addition, a compound parabolic concentrator was glued on the single-pixel detectors to collect the encoded light, which can make the system more compact in this case. In addition, another solution has been proposed recently using CS theory for chemical sensing applications; the encoding pattern was designed based on CS theory to reconstruct a higher-dimensional signal with fewer required numbers of measurements [133]. Figure 13b shows the detailed structure of the MEMS mask. It should be noted that an open row was set before all encoding patterns to realize self-triggering, and a gap (shown in the inset) could cause a dip in the measured signal when the gap entered the encoding row, so the mask dynamic positions could be located to determine correct mask patterns. Thus, the reported MEMS device gives the potential to construct a portable and truly miniaturized spectrometer for field applications with satisfactory pixel resolution. 

#### 3.4.2. Non-Visible Imaging

**Figure 14:**
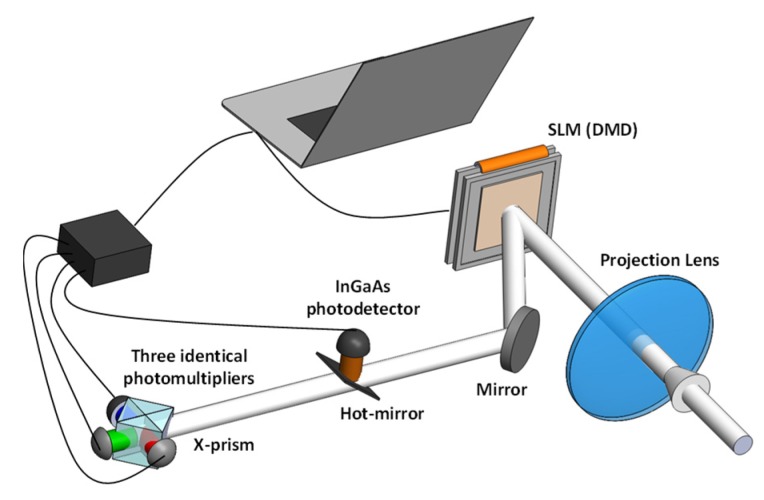
The schematic drawing of a DMD-based single-pixel imaging system to capture the visible images and infrared images at the same time [87].

Non-visible imaging systems have much significance in different sensing applications, as some unique properties can be obtained at a certain wavelength range. An infrared imager was reported to detect the leakage of methane gas using DMD [134] illuminated by a 1.651 um infrared laser diode. Sequential Hadamard encoding patterns were generated by the DMD with a pixel resolution of 16 × 16. This architecture was successfully used to capture methane gas leakage of 0.2 L/minute from a distance of 1 m. The framerate was reported to be 25 frame/s owing to the high switch speed of the DMD. Figure 14 depicts a single-pixel imager that was designed to capture the visible images and the short-wave infrared images at the same time [87]. Similar with the standard architecture shown in Figure 9, the scenery was focused onto the DMD where Hadamard encoding patterns were implemented. The encoded light passed through a hot-mirror to separate the short-wave infrared image from the encoded light beam. The infrared image was captured with InGaAs photodiode, while the visible images were recorded with visible red, blue and green channels after the images were separated with a dichroic prism. By utilizing the programmable ability of DMD, a fast optimization method was constructed to result in a framerate of 10 Hz in this case. CS theory was also incorporated with DMD-based infrared imager [135], and 64 × 64 pixels near-infrared images (830 nm) were captured from half of the required measurements. In addition, non-visible image capture has also been explored in multispectral imaging systems [136,137,138,139]. For example, the near-infrared images of colorful target objects were obtained in a polarimetric spectral imaging system [128]. In addition, DMD was also employed in other imaging fields to overcome the limitations of traditional imagers, such as photoacoustic microscopy systems [140]. A DMD-based photoacoustic motionless system was thus demonstrated to result in better performance than traditional systems, and its achievable axis range could achieve 1.8 m without sacrificing the resolution. It is noticed that a single-pixel imager obtains the potential to be operated for any wavelengths by conveniently changing the applied photodetector, but the properties of the applied SLM become a limiting factor for the achievable operating range of a specified imager. With special SLM, terahertz imaging can be achieved using the single-pixel imaging technology [92,141].

#### 3.4.3. Hyperspectral Imaging

**Figure 15:**
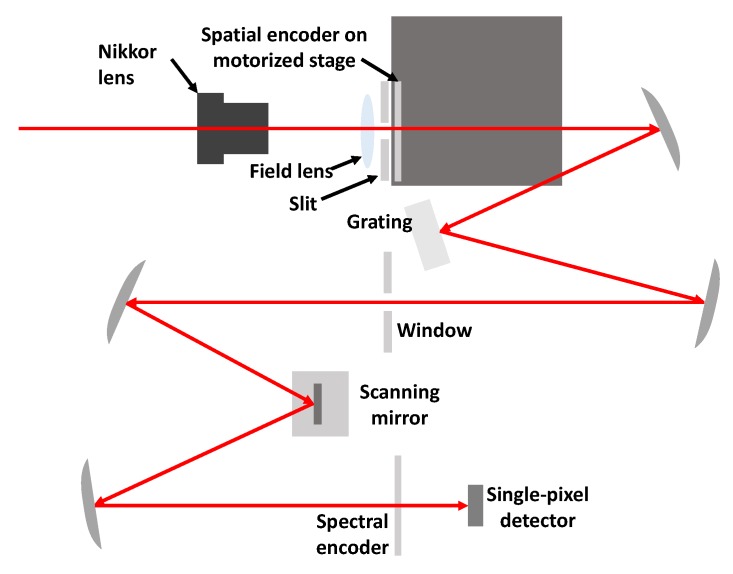
Schematic of a single-pixel hyperspectral imager in [149].

Hyperspectral imaging is a method to capture continuous 2D images over a wide spectral range, and thus specific properties of a 2D scenery can be extracted at a certain wavelength. The traditional way to construct a hyperspectral imager is to utilize mechanical slit scanning across the target or the object to capture its 3D information. This methodology requires a moving slit to realize the scanning motion, and limited amount of light that comes through the slit may affect the SNR. Thus, snapshot hyperspectral imagers were developed to capture 3D information in a single image. After the coded aperture was reported in a hyperspectral imager [142,143], computational technology became preferable in this field. Meanwhile, the DMD provides a more flexible choice in this field to replace the coded aperture in modulating spatial information. Single-pixel hyperspectral imaging systems can be found in [144,145,146,147,148]. Compressive sensing has also been explored to reduce the required number of measurements to speed up the acquisition time. However, it is noticed that the majority of hyperspectral imaging systems still utilizes sensor arrays to record the encoded signal. Thus, more recent research has been done by using the two separate encoding mechanisms to perform hyperspectral sampling, as shown in Figure 15 [149]. The incoming light was first modulated with an encoding mask before being dispersed to provide the spectral information. The encoded light containing spectral and spatial information was collected with a single-pixel detector, and a special Hadamard transform was conducted to perform the reconstruction. The applied resonant scanner can ensure a high operating speed, and the whole system could be constructed within a small package size

#### 3.4.4. Three-Dimensional Imaging and Lensless Imaging

**Figure 16:**
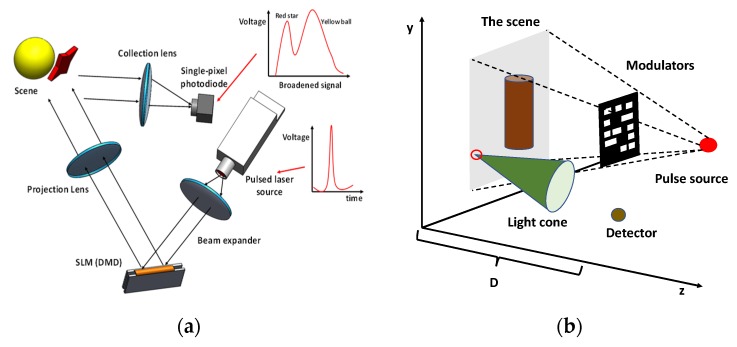
General architecture of (**a**) the time-of-flight approach to construct a 3D imaging with a pulsed laser source and high timing resolution detector [150] and (**b**) the lensless imaging system [157].

Another advantage associated with the encoding-based single-pixel imaging system is that the single-pixel detector can be fabricated with a very high timing resolution. With the advancement in technology, the time-of-flight approach has been developed to benefit the robotics, security and medical fields. Figure 16a shows a typical 3D imaging system using the time-of-flight approach, and such an imager can be found in [150], which gives a 10 Hz framerate using the Hadamard basis. When a short-pulsed light is supplied as the illumination light source, the signals captured by the detector are not only determined by the overlapping intensity between the encoding patterns and the scenery, but also the distance information. Since single-pixel detectors with picosecond response time are available, the distance information of each pixel can be distinguished using such detectors. From the given example, the reflected light from the red star and yellow ball is separately recorded by the detector. Thus, a 3D image can be constructed with the image of the scene and additional 2D depth information. It is worth mentioning that only one signal can be obtained with its corresponding encoding patterns in traditional 2D single-pixel imaging. In this case, one encoding pattern can generate a variety of measured signals by shifting the depth, thus befitting the development of a 3D data cube. Various research works have been devoted to this field [151,152,153]. Another method is to utilize more than one camera structure to capture the image of the scenery at different views or use different illumination patterns at one viewpoint. The performance of this 3D imager is typically determined by its viewpoints and the stereo vision geometry. Based on this concept, many single-pixel imaging systems have been reported for 3D imaging applications [154,155,156] with various mathematical models. It should also be noted that this system gets a stable depth resolution through an absolute measurement process, thus giving rise to the potential for long-distance measurements.

Similar to 3D imaging systems, single-pixel technology also befits the development of a potential imaging approach called lensless imaging. Figure 16b shows the typical architecture of lensless imaging systems, and no imaging and collection components are required in this case [157]. From the mentioned applications above, it should be noted that the imaging quality is highly affected by the properties of the applied optical components, especially when the system works in the non-visible range. For example, the aberrations of the lens may distort the resultant images. Using the power of computational technology, specific mathematical models can be established to describe the optical field after the scenery is modulated with special patterns, and a single-pixel detector is placed within its light field to collect a signal. Based on the measured signals and computational optics, the system can reconstruct a target image. Recently, a lensless imaging system was successfully demonstrated with an LCD device, which shows the potential of this technology [158]. With the development of sensor fabrication technology and computational technology, it has the potential to be a miniaturized imager.

## 4. Summary

In this paper, we review two main research directions for single-pixel imaging systems utilizing MEMS technology. The first methodology employs MEMS scanners to capture images, while the second method reconstructs the images through specified algorithms with the encoded signals. In terms of system configurations, the performance of the scanning systems is typically determined by the applied scanners. Four predominant actuation principles were reviewed, and each one has its potential and limitations. In general, higher scan angles and higher axial translation can increase the field-of-view of the imager, while higher frequency can increase its frame rate. Its power consumption is also an important factor for consideration. Electrostatic actuation has low power consumption despite the high voltage required, but the high voltage limits its applications. The comb-drive actuator is more commonly used compared to the parallel plate actuator as it has larger scan angles. Electromagnetic actuation can reach large scan angles with additional magnets, but this increases the package size and thus makes it less suitable for integration into imaging probes. PZT actuators can provide good scan frequency and scan angles, but its fabrication is more complex as the PZT film needs to be embedded within the beam actuators. Electrothermal actuation can obtain large scan angles and is more compact, but it consumes more power.Besides, the scan-based method can realize 3D imaging with the appropriate actuator design, such as in a focus-adjustable MEMS micromirror, while for the encoding-based approach, 3D images are usually obtained with the power of an applied detector and computational technology.

Considering the miniaturization, scan-based MEMS imagers can be constructed in a more compact size, but the field-of-view is limited compared with encoding-based MEMS imagers. Encoding-based MEMS imagers can also collect more light in each sampling process, thus the detected signal may be boosted, making it more desirable in long range imaging. In terms of cost, the SPI technology also provides the competitive edge over the detector array based camera in non-visible imaging applications, considering that the detector-array is expensive or unavailable in these wavelength ranges. It should be noted that although the detected signal is increased, the encoded information is affected by the detector noise, which varies about a mean value. Thus, to ensure the recovered image quality, the detector and light source have to be stable during the acquisition process. Regarding the applications of these two methodologies, scanning-based single-pixel imaging systems see major use in the biological imaging field. In this field, MEMS technology provides an efficient way to construct a miniaturized imager for different applications. While it may be quite difficult to further integrate a DMD-based imager, it benefits from both single-pixel imaging technology and computational technology, thus providing competitive edges over the traditional scanning method in terms of sampling time, but the post processing required may be longer than the scan-based method due to the computational overhead.

Overall, the scan-based MEMS system is widely expected to be a highly useful tool in biomedical imaging applications in the future, owing to its ability for miniaturization, while encoding-based MEMS systems have the potential for long range imaging, 3D imaging and lensless imaging due to the rapid advancement of single-pixel detector technology and computational technology.

## Figures and Tables

**Table 1:** A summarized table of various modulators applied in single-pixel imaging systems.

Modulators	Compactness and Illumination	Modulation Efficiency	Pixel Resolution and Availability	Operating Wavelengths
Digital micromirror device (DMD)	Miniature device; active/passive illumination.	High speed; simple modulation; programmable.	Flexible pixel resolution; commercially available.	Wide range; micromirrors determined.
(Liquid crystals on sicon(LCOS)/Liquid crystal devices(LCD)	Miniature device; active/passive illumination.	Slow speed; modulate the phase and amplitude of the light; programmable.	Flexible pixel resolution; commercially available.	Wide range; liquid crystals determined.
Customized diffuser	Poor compactness; active/passive illumination.	Slow speed; complicated modulation; not programmable.	Fixed pixel resolution; customized fabrication; costly.	Wide range; materials determined.
Mechanical mask	Can be miniaturized; active/passive illumination.	Slow speed; simple binary modulation patterns; not programmable.	Fixed pixel resolution; customized fabrication; cheap.	Wide range; materials determined.
optical phase arrays (OPA)	On the process of miniaturization; active/passive illumination.	High speed; simple random patterns; controllable.	Flexible pixel resolution; complicated fabrication; costly.	Limited narrow range; light-emitting components determined.
light-emitting diode (LED) arrays	On the process of miniaturization; active illumination.	High speed; simple binary patterns; programmable.	Flexible pixel resolution; commercially available.	Limited narrow range; light-emitting components determined.

**Table 2:** A summarized table of three commonly applied sampling strategies in single-pixel imaging system.

Sampling Strategy	Prior Knowledge	Sampling Speed	Computational Complexity
Orthogonal sampling	No prior knowledge; no signal representation.	Full measurements; slow sampling speed.	Simple computation.
Sub-orthogonal sampling	A specific prior knowledge; no signal representation.	Compressed measurements; high sampling speed.	Not computationally demanding.
Compressive sensing	A general sparse assumption; requires sparse representation.	Compressed measurements; high sampling speed.	Computational overhead.
